# Antitumor therapy for breast cancer: Focus on tumor‐associated macrophages and nanosized drug delivery systems

**DOI:** 10.1002/cam4.5489

**Published:** 2023-02-16

**Authors:** Cuiping Zhan, Ying Jin, Xinzhi Xu, Jiangbo Shao, Chunxiang Jin

**Affiliations:** ^1^ Department of Ultrasound China‐Japan Union Hospital of Jilin University Changchun China; ^2^ Department of Breast Surgery The First Hospital of Jilin University Changchun China; ^3^ Department of Ultrasound Chongqing University Cancer Hospital Chongqing China

**Keywords:** breast cancer, macrophages, nanotechnology, target therapy

## Abstract

**Background:**

In breast cancer (BC), tumor‐associated macrophages (TAMs) are an important component of the tumor microenvironment and are closely related to poor prognosis. A growing number of studies have focused on the role of TAMs in BC progression and therapeutic strategies targeting TAMs. As an emerging treatment, the application of nanosized drug delivery systems (NDDSs) in the treatment of BC by targeting TAMs has attracted much attention.

**Aims:**

This review is to summarize the characteristics and treatment strategies targeting TAMs in BC and to clarify the applications of NDDSs targeting TAMs in the treatment of BC by targeting TAMs.

**Materials & Methods:**

The existing results related to characteristics of TAMs in BC, BC treatment strategies by targeting TAMs, and the applications of NDDSs in these strategies are described. Through analyzing these results, the advantages and disadvantages of the treatment strategies using NDDSs are discussed, which could provide advices on designing NDDSs for BC treatment.

**Results:**

TAMs are one of the most prominent noncancer cell types in BC. TAMs not only promote angiogenesis, tumor growth and metastasis but also lead to therapeutic resistance and immunosuppression. Mainly four strategies have been used to target TAMs for BC therapy, which include depleting macrophages, blocking recruitment, reprogramming to attain an anti‐tumor phenotype, and increasing phagocytosis. Since NDDSs can efficiently deliver drugs to TAMs with low toxicity, they are promising approaches for targeting TAMs in tumor therapy. NDDSs with various structures can deliver immunotherapeutic agents and nucleic acid therapeutics to TAMs. In addition, NDDSs can realize combination therapies.

**Discussion:**

TAMs play a critical role in the progression of BC. An increasing number of strategies have been proposed to regulate TAMs. Compared with free drugs, NDDSs targeting TAMs improve drug concentration, reduce toxicity and realize combination therapies. However, in order to achieve better therapeutic efficacy, there are still some disadvantages that need to be considered in the design of NDDSs.

**Conclusion:**

TAMs play an important role in the progression of BC, and targeting TAMs is a promising strategy for BC therapy. In particular, NDDSs targeting TAMs have unique advantages and are potential treatments for BC.

## Graphical abstract

TAMs come from either bone marrow or the yolk sac. According to the polarized phenotype, there are two main types of macrophages, M1‐type, which exerts pro‐inflammatory and anti‐tumor effects, and M2‐type, which exerts anti‐inflammatory and protumor effects. The major strategies used to target TAMs for BC therapy include depleting macrophages, blocking recruitment, reprogramming to attain an anti‐tumor phenotype, and increasing phagocytosis. NDDSs can carry a variety of therapeutic reagents to target TAMs and realize combination therapies in BC.

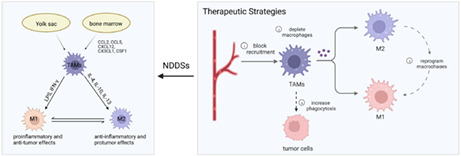

## 1 INTRODUCTION

BC is one of the most common malignant tumors, with an incidence of 24.5% and mortality of 15.5%.^1^ BCs are classified into four types including Luminal A, Luminal B, human epidermal growth factor receptor (HER) 2+, and triple negative breast cancer (TNBC) according to the different expression of estrogen receptor (ER), progesterone receptor (PR), HER2, and Ki67.^2^ The treatment of BC depends on its molecular classification. Despite significant progress in the target and endocrine therapy of BC in recent years, the prognosis of patients with BC is still unsatisfactory due to the high frequency of recurrence and metastasis. Recent evidence has shown that the tumor immune microenvironment (TIME) has an important effect on the occurrence and development of BC.^3^ BC is reported as an immune "cold" with the lack of infiltration of immune cells and an inherent immunosuppressive microenvironment.^4^ This microenvironment facilitates immune evasion of tumor cells and thus leads to high tumor recurrence and metastasis rate, which limits the effects of radiation, chemotherapy, or immunotherapy for BCs.

TAMs are an important component of TIME and account for 50% of the tumor mass.^5^ Current evidence indicates that TAMs engage in complex interactions with cancer cells, natural killer cells, T cells, endothelial cells, and fibroblasts, and they are recognized as critical players promoting tumor growth, metastasis, and angiogenesis.^6^, ^7^, ^8^ Moreover, it has been suggested that TAMs induce resistance to chemotherapy and are associated with poor overall survival (OS) in patients with BC.^9^ The therapies targeting TAMs can enhance the antitumor effect and relieve immunosuppression by altering the immunosuppressive microenvironment.^10^ Therefore, such therapies may become a promising strategy in the treatment of BC.

In the past few years, many drugs have been found with the ability to affect TAMs directly and/or indirectly.^11^ However, the practical applications are still far from satisfactory due to the solubility, pharmacokinetics, and systemic side effects. To deal with this issue, a wide variety of NDDSs have been used to deliver specific therapeutic agents directly to the tumor sites and control drug release by the smart response.^12^ Nanoparticle structures can facilitate the delivery of drugs to the tumor tissues, as well as regulate the function of TAMs, thus improving the treatment of BCs. In this paper, we review the characteristics of TAMs in BCs, therapeutic strategies against TAMs, and applications of NDDSs in TAM‐specific drugs, in order to provide a comprehensive understanding and potential treatment strategies for improving the treatment efficacy of BCs.

## 2 TAMS IN BC

### 2.1 origin and subtypes of TAMs

**FIGURE 1:**
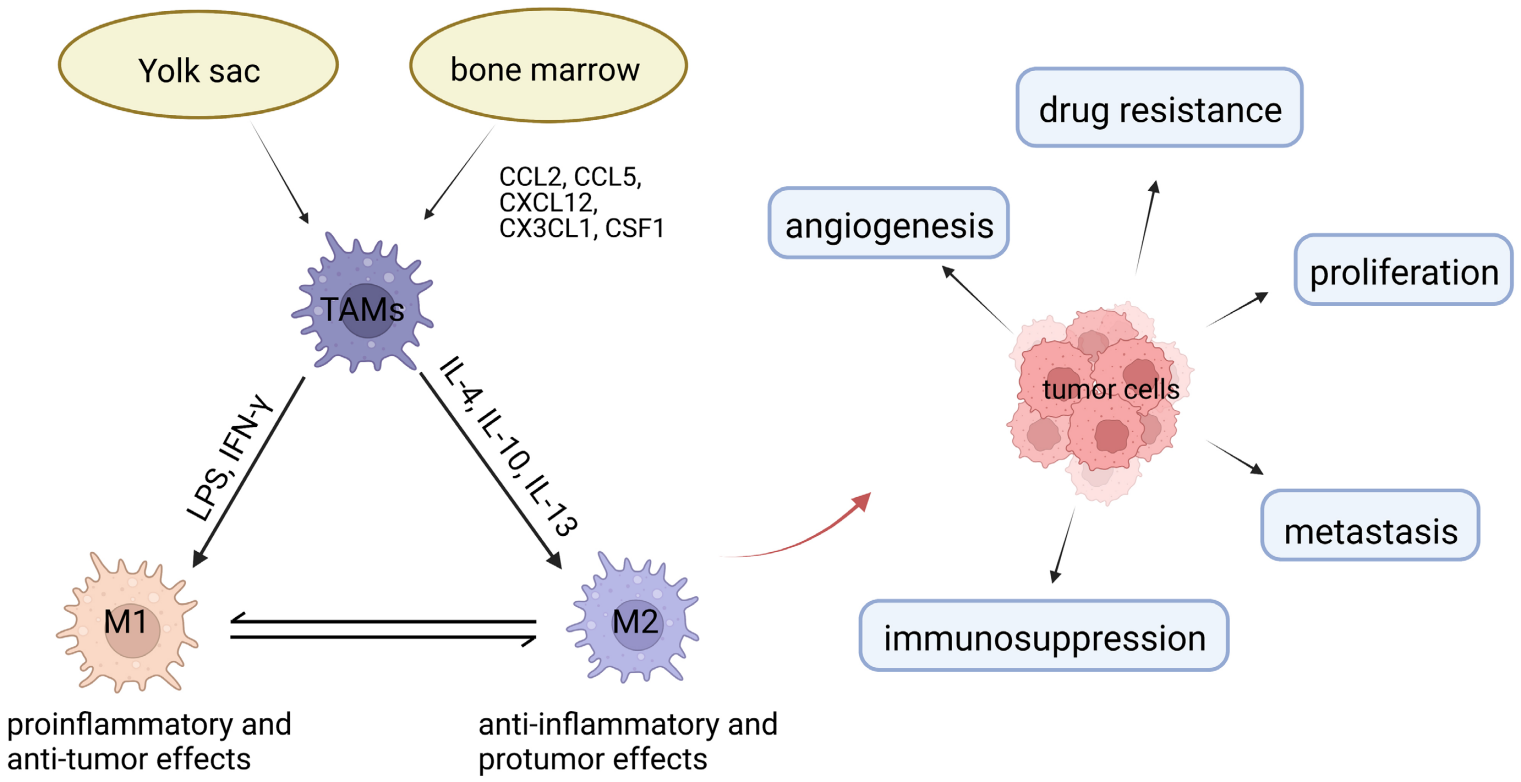
The origin, subtypes, and functions of TAMs in BC. TAMs come from bone marrow or the yolk sac. There are two major phenotypes of TAMs: M1 and M2. M1‐type macrophages exert pro‐inflammatory and anti‐tumor effects. M2‐type macrophages have anti‐inflammatory and pro‐tumor functions. TAMs, especially M2‐type macrophages, promote tumor cell proliferation, metastasis, angiogenesis, immunosuppression, and drug resistance in BC.

The tumor microenvironment (TME) is composed of multiple types of cells (immune cells, endothelial cells, cancer stem cells, fibroblasts, etc.) as well as cellular components (cytokines, chemokines, extracellular matrix, etc.).^13^ Macrophages are one of the most prominent tumor‐associated noncancer cell types in TME.^5^ There are pieces of evidence showing that TAMs come from either bone marrow or the yolk sac.^14^ TAMs are heterogeneous in BC, with a wide variety of polarized phenotypes. According to the polarized phenotype, there are two main types of macrophages, M1‐type, which is considered as classical macrophages, and M2‐type, known as alternatively activated macrophages.^15^, ^16^, ^17^ The M1‐type macrophages, which are stimulated by lipopolysaccharide (LPS) and the type 1 T helper cell (Th1) cytokines, have strong cytotoxicity and phagocytosis to tumor cells and exert pro‐inflammatory and anti‐tumor effects.^18^ Conversely, the M2‐type macrophages, which are induced by the type 2 T helper cell (Th2) cytokines, such as interleukin (IL)‐4, IL‐10, or IL‐13, promote angiogenesis, tumor growth, and metastasis.^18^ In vivo, there is a dynamic balance between M1‐type and M2‐type macrophages. The balanced state of TAMs can affect cancer progression and outcomes in BC.^19^, ^20^ The origin, subtypes, and functions of TAMs in BC are shown in Figure 1.

### 2.2 Functions and prognosis of TAMs

TAMs play a key role in the progression of BC. After being recruited into BC tissues, macrophages mediate tumor cell proliferation, metastasis, angiogenesis, and immunosuppression through a wide variety of mechanisms. Studies have shown that macrophages promote the up‐regulation of genes associated with malignant biological behavior in tumor cells in vitro.^21^ Further research showed that TAMs contribute to tumor growth, metastasis, angiogenesis, and immunosuppression by secreting various cytokines.^22^ The IL secreted by TAMs, such as IL‐6, IL‐8, and IL‐10, can promote the proliferation and metastasis of tumor cells, as well as enriches the cancer stem cell population.^23^, ^24^, ^25^ CC‐chemokine ligand (CCL) 2 and CCL18 released by TAMs could markedly elevate the EMT, invasion, and migration events in BC cells.^24^, ^26^ Moreover, TAMs can be involved in angiogenesis by producing a series of pro‐angiogenic factors, including vascular endothelial growth factor (VEGF), SEMA and S100A families.^27^ The immunosuppressive effect of macrophages is mainly manifested in two aspects. First, signal regulatory protein α (SIRPα) expressed on macrophages can bind to CD47, which is highly expressed on tumor cell membranes, to enhance the "don't eat me" signal.^28^ Similarly, CD24 / sialic‐acid‐binding Ig‐like lectin 10 (Siglec‐10) axis and programmed cell death protein 1 (PD‐1) / programmed death ligand‐1 (PD‐L1) axis also attenuate phagocytosis of macrophages and promote tumor cell escape.^29^, ^30^ Secondly, TAMs inhibited T cell function through expressing PD‐L1 and secreting anti‐inflammatory cytokines.^31^ TAM‐secreted CXCL1, CCL22, and CCL17 have been proven to induce the recruitment and differentiation of Tregs in BC tissues.^32^, ^33^ Overall, TAMs play a dual role in promoting tumor progress and suppressive immune microenvironment.

Moreover, several studies have reported the negative correlation between TAMs and the response to chemotherapy in patients with BC.^34^, ^35^ TAMs can induce drug resistance in BC by secreting various cytokines, such as CCL2, IL‐6, and transforming growth factor‐beta1 (TGFβ1).^36^ The CCL2 secreted by TAMs can induce tamoxifen resistance through PI3K/Akt/mTOR signal transduction.^37^ IL‐6 secreted by TAMs contributes to adriamycin resistance in luminal BC.^38^ Hepatic leukemia factor, which is regulated by TGFβ1 secreted by TAMs, transactivated gamma‐glutamyltransferase 1 and drove TNBC cell cisplatin resistance.^39^ Therefore, targeting TAMs is a potential strategy for overcoming drug resistance in BC.

The contribution of TAMs to cancer outcomes is multifaceted due to different polarizing phenotypes. Recent evidence has shown that the infiltration of M2‐type TAMs both in the TN and TS is related to a significantly higher risk of aggressive features, and is an independent prognostic factor of OS in patients with TNBC.^35^, ^40^, ^41^ However, patients with a high proportion of M1‐type macrophages showed less advanced disease and better patient outcomes.^42^ There are similar findings in HER2+ BC. A high number of inducible nitric oxide synthase (iNOS)+ M1‐type macrophages in the invasive margin and the center of the tumor is significantly associated with improved survival.^43^ In all, a high proportion of M2/M1 macrophages are clinically related to poor outcomes in BC. TAMs, especially the M2 phenotype, may be a prognostic biomarker of BC.

### 2.3 Biomarkers of TAMs

Due to the above facts, TAMs can be targeted to treat BC, that is, to reduce the proportion of M2‐type macrophages and increase the proportion of M1‐type macrophages. To verify the effect of treatments, the proportion of M1‐type and M2‐type macrophages in TME needs to be verified. A lot of biomarkers that can specifically identify TAMs have been found. The most common biomarkers of TAMs are transmembrane receptors.^44^ Other than that, multiple new biomarkers that allowed a more accurate description of the phenotypic and functional characterizer of TAMs have been reported recently, including cytokines, enzymes, transcription factors, and so on. As preclinical experiments are commonly performed in mouse models, it is important to understand the biomarkers of TAMs in humans and mice. Some explanations regarding the differences between them are provided in the following. Human macrophages are identified by high expression of CD68, while murine macrophages are distinguished by high expression of F4/80.^45^ In human BC, the biomarkers of M1‐type TAMs include CD80, CD86, iNOS, human leukocyte antigen‐DRα (HLA‐DRα) and YKL‐40, IL‐1β, IL‐6, IL‐12, TNFα, and main biomarkers of M2‐type TAMs are CD206, CD163, CD204, stabilin‐1, folate receptor beta (FRβ), arginase‐1 (Arg‐1), YKL‐39, IL‐4, IL‐10, IL‐13, CCL2, CCL18, TGFβ, and VEGF.^26^, ^27^, ^36^, ^44^, ^46^, ^47^, ^48^, ^49^, ^50^, ^51^, ^52^ Some biomarkers of macrophage polarization in murine BC are different from those in humans. It is known that HLA‐Drα and Ykl39 are not expressed on TAMs of murine BC.^45^ On the contrary, Fizz1 and Ym1 are induced by IL‐4 and IL‐13, while they are not expressed in human TAMs.^45^ The expression of CD204 and FRβ remains to be confirmed in M2‐type TAMs of murine BC. The biomarkers of TAMs are shown in Table 1.

**TABLE 1:** The biomarkers of TAMs in human and murine BC

	M1	M2
Human BC	CD80, CD86, HLA‐DRα, iNOS, YKL‐40, IL‐1β, IL‐6, IL‐12, TNFα	CD206, CD163, CD204, stabilin‐1, FRβ, Arg‐1, YKL‐39, IL‐4, IL‐10, IL‐13, CCL2, CCL18, TGFβ, VEGF
Murine BC	CD80, CD86, iNOS, YKL‐40, IL‐1β, IL‐6, IL‐12, TNFα	CD206, CD163, stabilin‐1, Arg‐1, Fizz1, Ym1, IL‐4, IL‐10, IL‐13, CCL2, CCL18, TGFβ, VEGF

### 2.4 Location in different BCs

BC is a heterogeneous tumor, and BCs in different molecular types show very different TAMs profiles.^53^ The infiltration of macrophages was more common in TNBC than in non‐TNBC.^54^ High density of CD163+ TAMs was founded associated with most of TNBC. However, luminal A tumors were accompanied by low levels of CD163+ TAMs in both tumor nest and tumor stroma.^55^ ER+ BC and TNBC induce the transformation of macrophages into different phenotypes and functions.^21^ The more aggressive MDA‐MB‐231 cells promote monocyte differentiation into M2‐type macrophages, while T47D cells induced a pro‐inflammatory and anti‐tumor phenotype.^21^, ^56^, ^57^ It can be seen that TNBC is characterized with unique TAM subsets, which differs from luminal subtypes. The ability to polarize macrophages to M2 phenotype appears to be a characteristic of basal but not luminal cells, and this may explain why high infiltration of macrophages in TNBC tumors is associated with poor prognosis.

## 3 THERAPEUTIC STRATEGIES TARGETING TAMS FOR BC

**FIGURE 2:**
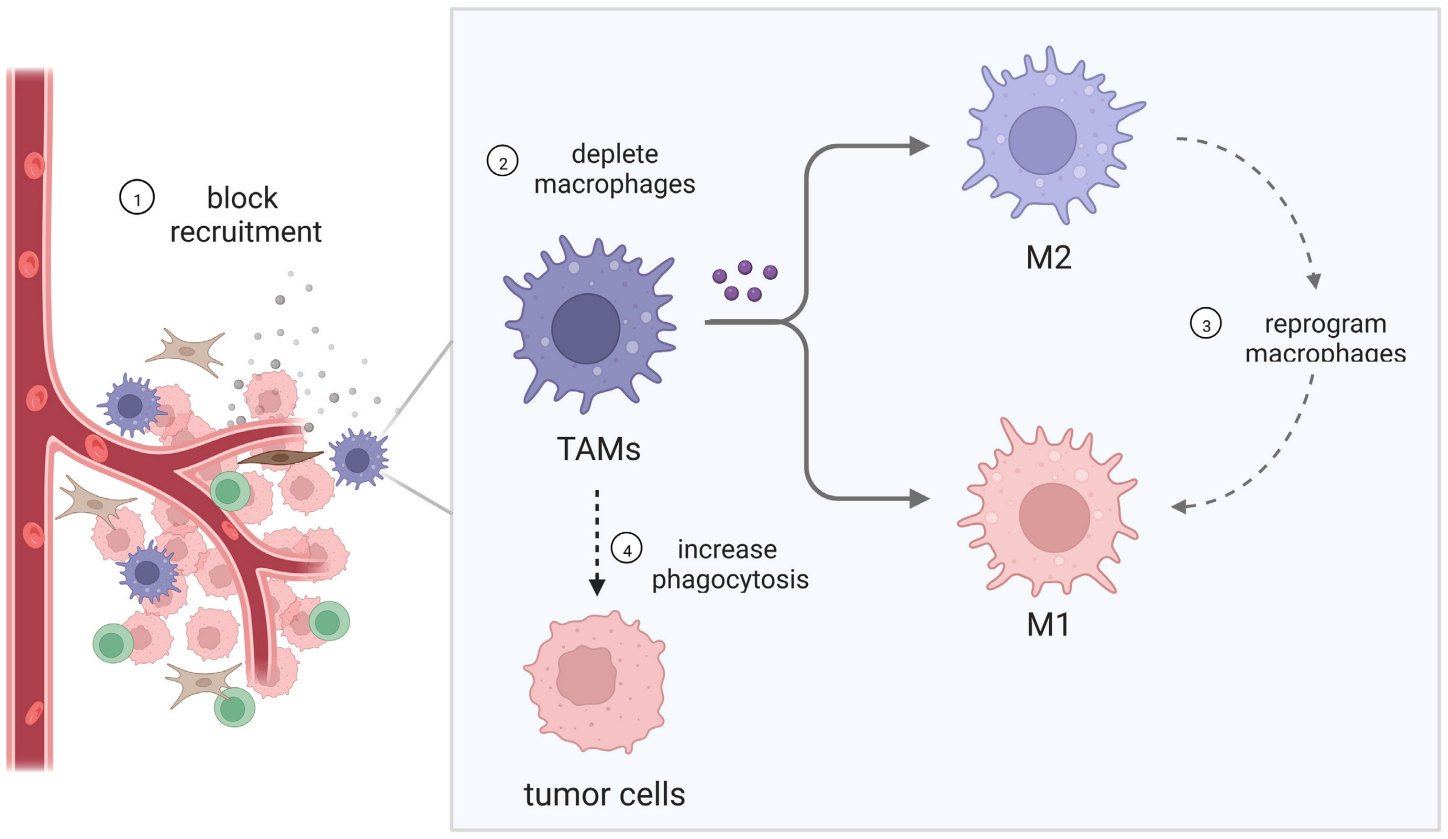
The major strategies used to target TAMs for breast cancer therapy.

Due to the effects of TAMs on BC growth, metastasis, and drug resistance, great progress has been made in TAM‐targeted therapies in the past few years.^11^ The major strategies used to target TAMs for BC therapy include depleting macrophages, blocking recruitment, reprogramming to attain an anti‐tumor phenotype, and increasing phagocytosis (Figure 2).

### 3.1 Depleting macrophages

TAMs in BC are primarily a subpopulation with an M2 phenotype and exert pro‐tumor function.^44^ One way to limit the effects of TAMs is to reduce the number of TAMs and control macrophage proliferation. By now, clodronate has been the important choice for TAMs depletion.^58^ In order to improve the pharmacokinetics of clodronate, clodronate liposomes are used to deplete TAMs and reduce tumor volumes and weights in a 4T1 mouse model.^59^ Meanwhile, colony‐stimulating factor 1 receptor (CSF1R) is highly expressed in macrophages and controls cell survival, proliferation, and differentiation.^59^ Blockade of CSF1R by monoclonal antibodies or small molecule inhibitors can deplete TAMs and increase the percentage of intratumoral T cells.^59^ However, these blockers not only target M2‐type macrophages but also affect the activity of M1‐type macrophages. Thus, the selective depletion of M2‐type macrophages needs further study.

### 3.2 Blocking macrophage recruitment

Given that monocytes/macrophages recruit from the blood and infiltrate tumor tissues, it is necessary to identify and block factors that induce macrophage recruitment. In BC, studies have shown that the recruitment of macrophages is regulated by a variety of cytokines, such as CCL, chemokine (C‐X‐C motif) ligand (CXCL), and colony‐stimulating factor 1 (CSF1).^54^, ^60^, ^61^ In the following, we describe the important role of these cytokines in the recruitment of macrophages and promotion of BC progression and the potential as targets for the treatment of BC.

The CCL family includes multiple members among which CCL2 and CCL5 play a key role in the recruitment of macrophages. Next, we introduce the roles of these two cytokines in BC progression and the therapeutic potentials of their corresponding neutralizing antibodies. CCL2, also known as monocyte chemotactic protein 1 (MCP‐1), is synthesized by tumor cells, tumor‐associated mesenchymal stromal cells (TA‐MSCs), and cancer‐associated fibroblasts (CAFs).^60^, ^62^, ^63^, ^64^ It promotes angiogenesis and enhances BC metastasis to lung and bone through recruiting monocytes which express C‐C chemokine receptor type 2 (CCR2).^13^ In addition to recruiting macrophages, CCL2 also promotes the recruitment of Tregs with high CCR2 expression.^65^ The overexpression of CCL2 is more likely to form a tumor immunosuppressive microenvironment. Meanwhile, the relationship between CCL2 expression and the clinical characteristics of patients was investigated.^66^ The expression of CCL2 was negatively related to the overall stage, but not related to tumor grade, ER, PR, or HER2 status in patients with BC. Although CCL2 plays an antitumor role in tumor tissue, current studies suggest that it is not associated with OS in patients with BC. Based on these findings, CCL2‐neutralizing antibodies are used to treat BC in preclinical animal models and clinical trials. In the murine BC model, delivery of CCL2‐neutralizing antibody inhibited monocyte release from bone marrow, macrophage infiltration, and vascular infiltration, thereby reducing the formation of lung metastases.^67^ However, it was found that discontinuation of CCL2‐neutralizing antibodies models led to an overshoot of metastases in BC.^67^ In addition, clinical trials have reported a temporary suppression of free CCL2 levels in solid tumors after administration of the CCL2‐neutralizing antibody (CNTO888), followed by an increase in free CCL2 concentrations even beyond pre‐treatment baseline levels.^68^ The main reason may be that CCL2‐neutralizing antibodies cannot effectively block the CCL2‐CCR2 axis for a long enough time. Antibodies are cleared within 10 days after treatment in vivo, resulting in the rebound of the CCL2 level.^67^ Due to this fact, CNTO888 has shown limited efficacy as a single agent or in combination with chemotherapy in the treatment of solid tumors (NCT00537368, NCT01204996).^68^, ^69^ Anti‐CCL2 drugs need to be used with caution for solid tumors.

CCL5 is another important member of CCL that can elevate macrophage infiltration.^70^, ^71^ Several studies have found that tumor‐derived CCL5 can bind to CCR5 expressed on macrophages.^70^, ^72^, ^73^, ^74^ The activation of CCR5 can further stimulate STAT3 and AKT signaling to promote macrophage recruitment and M2‐type polarization.^70^, ^71^ Recruited macrophages by CCL5 have been shown to secrete collagen and collagen deposition factors and promoted tumor recurrence.^70^ In addition to its effect on macrophages, CCL5 can also affect the recruitment and differentiation of T lymphocytes. CCL5 promoted the differentiation of Th2 cells by activating CCR3 and boosting Gfi1 expression in TME. This phenomenon is evident in patients with advanced BC.^75^ The effect of CCL5 on BC progression is two‐sided. CCL5 secreted by tumor cells promotes the recruitment of CD8+ T cells and plays an antitumor role. Ozga et al.^76^ consider that the balance between these different functions may depend on the stage of tumorigenesis, the state of immune cell activation, and the relative expression of chemokine receptors. These factors still need to be further clarified by in vitro and in vivo experiments. Analysis of clinical samples showed that the expression of CCL5 in BC tissues was higher than that in adjacent normal tissues, and was related to the stage of BC and lymph node metastasis. Studies have shown that CCL5 overexpression is associated with poor disease‐free survival in BC.^77^, ^78^ However, there was a significant correlation between overexpression of CCL5 and increased OS in patients with TNBC.^79^ This may be related to the infiltration of immune cells in the tumor tissue. Immune cell infiltration in TNBC is different from other subtypes of BC. TNBC shows higher numbers of tumor‐infiltrating lymphocytes (TILs) compared to ER+ tumors.^80^ TILs are one of the important sources of CCL5 in TME, and TIL count has a positive correlation with CCL5 in TNBC.^81^ It is reported that a high expression of TILs is associated with a good outcome.^81^ This is consistent with the relationship between CCL5 and prognosis in TNBC. Therefore, when discussing the relationship between CCL5 and the prognosis of BC, the degree of lymphocyte infiltration should not be ignored. Given the dual role of CCL5 in BC, the researchers suppressed CCL5 expression in mice to observe the therapeutic effect. In the murine ER+ BC model, the use of an anti‐CCL5 neutralizing antibody significantly decreases the infiltration of macrophages and tumor volume.^73^ Further studies are needed to evaluate the efficacy of anti‐CCL5 neutralizing antibody in different molecular subtypes and clinical stages of breast cancer.

The Chemokine CXC subfamily is a vital regulator of the recruitment of TAMs. CXCL12, belonging to the chemokine CXC subfamily, is released by stromal cells and fibroblasts in BC.^82^ It can attract cells expressing Chemokine (C‐X‐C motif) receptor 4 (CXCR4).^82^ Through the release of CXCL12, the transcription factor CXCR class 1 homeobox 1 in tumor cells, also known as Pit‐1, was found to mediate the recruitment and polarization of macrophages.^61^ Moreover, high CXCL12 expression in patients with basal‐like BC is associated with high an accumulation of Tregs in tumors.^83^ Treatment of BC by using CXCL12 antibody significantly decreased CD163 and VEGFA mRNA expression in TAMs, resulting in decreased M2‐type macrophages and angiogenesis.^61^ Preclinical and clinical studies are needed to confirm the benefit of blocking the CXCL12/CXCR4 axis in patients with BC. CX3CL1 is another member of the chemokine CXC subfamily, which can bind to CX3CR1 that is highly expressed on macrophages, and increase the accumulation of macrophages in tumor tissues.^84^ iFGFR1‐induced CX3CL1 enhanced the migration of macrophages during the initial stage of tumor formation and blocking CX3CR1 significantly decreased the recruitment of macrophages in MMTV‐iFGFR1 mice.^84^ Dreyer et al.^85^ also found that CX3CL1 deficiency delayed mammary tumorigenesis in Tg‐neu mice. However, CX3CL1 plays a protective role in established BC models. Overexpressed CX3CL1 attracted CD3‐CD49b+ NK cells, CD3+CD4+ T cells, and CD3+CD8+ cytotoxic T cells to tumor tissues in the 4T1 animal model, whereas regulatory T cells, F4/80+MHC II+ macrophages, and CD11b+ cells did not significantly change.^85^ The overexpression of CX3CL1 inhibited tumor growth and lung metastasis in vivo.^85^ Thereby, the effect of CX3CL1 on tumor progression is completely different in different stages of BC. Further studies are needed to understand the complex role of CX3CL1 in BC and its potential for BC treatments.

By now, there are reports indicating that CSF1 produced by BC cells promotes macrophage recruitment through binding to CSF1R.^54^, ^86^ The results of immunohistochemistry showed that the expression of CSF1 correlates with marked CD68+ monocytes infiltrates and prognosis in primary breast adenocarcinomas.^87^ The secretion of CSF1 is different in different BCs. The mean CSF1 level was much higher in MDA‐MB‐231 and MDA‐MB‐468 conditioned medium compared to that of MCF‐7.^88^ The inhibition of CSF1/CSF1R pathway in MDA‐MB‐231 abrogates macrophage infiltration and consequently reduces tumorigenesis in vitro and in vivo.^88^ In the mouse mammary tumor virus‐driven polyomavirus middle T antigen (MMTV‐PyMT) model of mammary carcinogenesis, using BLZ945, a small molecule inhibitor of CSF1R, significantly decreased the number of macrophages and increased the number of CD8+ T cells in tumor tissues, leading to attenuate the growth of the primary mammary tumor.^86^ Blocking the CSF1‐CSF1R pathway is essential to inhibit macrophage recruitment to tumor tissues. However, several clinical trials to inhibit CSF1R have failed to achieve the desired results. In advanced solid tumors, anti‐CSF1R emactuzumab did not translate into objective clinical responses either as monotherapy or in combination with paclitaxel or CD40 agonist (selicrelumab).^89^, ^90^ More clinical studies targeting CSF1R are ongoing. The feasibility of blocking the CSF1/CSF1R pathway remains to be demonstrated.

### 3.3 Reprogramming macrophages

The conversion between the different polarization phenotypes of TAMs is influenced by TME. A variety of factors and pathogenic signals produced by tumor cells and other cells can promote the TAMs to polarize into M2‐type macrophages and promote tumor growth and metastasis. Herein, we summarize the factors that promote macrophages to M2 polarization in BC tissues and the changes of related signaling pathways in TAMs.

#### 3.3.1 TME and macrophage polarization

TAMs expose to the complex microenvironment of BC. Signals originating from tumor cells, lymphocytes, CAFs, and matrices cooperatively regulate the heterogeneity and function of TAMs. In the following, we introduce the effects of cells and matrix on macrophage polarization in TME.

Evidence shows that tumor cells educate macrophages toward the M2 activation status through secreting cytokines and lactic acid, which is conducive to their proliferation, invasion, and migration. Cytokines involved in macrophage recruitment, including CCL2, CCL5, CXCL12, and CSF1, have also been confirmed to promote M2 polarization of TAMs.^74^, ^91^, ^92^, ^93^ In addition to that, tumor cell‐derived TGFβ can promote M2 polarization by suppressing transcription factor EB (TFEB) activation and expression in macrophages.^94^ Polarized macrophages by TGF‐βplayed a role in promoting tumor growth. Reprogramming of energy metabolism is a hallmark of BC.^95^ Tumor cells preferentially undergoing glycolysis rather than oxidative phosphorylation, even under the condition of normal oxygen, is favorable for the production of lactic acid.^96^ Preclinical evidence showed that tumor‐derived lactic acid in TME induces M2‐type polarization via the activation of the HIF‐1α/STAT3, ERK/STAT3, and PKA/CREB signaling pathway in BC.^97^, ^98^, ^99^ Lactate‐stimulated M2‐type polarization induced BC cell proliferation, migration, angiogenesis, and tamoxifen resistance.^97^, ^98^, ^99^

Increasing evidence reveals that tumor‐derived exosomes are required for the regulation of macrophage polarization during the progression of BC.^100^ Exosomes contain a large number of functional microRNAs (miRNAs). It has been confirmed that several miRNAs expressed in tumor cells are delivered to macrophages by exosomes and regulate macrophage polarization. MiR‐138‐5p is a miRNA that was overexpressed in exosomes isolated from MDA‐MB‐231 cells or T47D cells. MiR‐138‐5p inhibited M1 polarization and promoted M2 polarization by inhibiting the expression of KDM6B in macrophages.^101^ In turn, macrophages treated with exosome miR‐138‐5p promoted lung metastasis of BC. MiR‐222 is another miRNA in exosomes that can regulate macrophage polarization. MiR‐222 was highly expressed in exosomes from adriamycin‐resistant MCF‐7 cells.^102^ After entering macrophages, miR‐222 induced M2 polarization of macrophages through the PTEN/Akt pathway, which promoted the proliferation, migration, and invasion of BC cells. Therefore, changing the expression of miRNAs in exosomes can reprogram macrophages to exert antitumor effects. Jiang et al.^103^ found that epigallocatechin gallate (EGCG) suppressed breast tumor growth by inhibiting tumor‐associated macrophage infiltration and M2 polarization. EGCG up‐regulated miR‐16 in tumor cells, which can be transferred to TAM via exosomes and active NF‐κB pathway. These data suggested that miRNAs in exosomes can influence breast tumor growth by regulating TAMs polarization and can be potential therapeutic targets for BC.

Th1 and Th2 cells secreted Th1 and Th2 cytokines, respectively. Th1 and Th2 cytokines have different functions on macrophage polarization. Th2 cytokines, such as IL‐4, IL‐6, and IL‐13, have been reported to contribute to M2 polarization of TAMs and BC metastasis. IL‐4 or IL‐13 mediated phosphorylation of STAT6 (Tyr641) and induced the M2 polarization of macrophages in inflammatory BC.^104^ Blocking IL‐4 and IL‐13 can decrease the number of M2‐type macrophages and protect against radioresistance of inflammatory BC. IL‐6 was found to mediate M2 polarization through the mTORC2‐Akt1 axis and increased distant metastasis.^105^ Different from Th2, Th1 cytokines have been proven to polarize macrophages into M1 phenotype. Interferon‐gamma (IFN‐γ) is one of the Th1 cytokines. Sun et al.^106^ have demonstrated that the combined usage of Monophosphoryl lipid A and IFN‐γ can reprogram CD206+ TAMs to iNOS+ macrophages. The reprogrammed macrophages secreted IL‐12 and tumor necrosis factor‐α (TNFα) to activate cytotoxic T cells. This systemic anti‐tumor immune response reduced the growth and metastasis of PyMT or 4T1 breast tumors.

Crosstalk between cancer‐associated fibroblasts (CAFs) and TAMs can mediate the polarization of macrophages and tumor progression. A recent study showed that CAFs were associated with CD163+ macrophage infiltration in patients with TNBC.^107^ Yavuz et al.^63^ reported that CAFs recruited monocytes and induced M2 polarization of TAMs by secreting CCL2 and stromal cell‐derived factor‐1 (SDF‐1). The polarized TAMs exerted immunosuppressive effects through the PD‐1 axis. In addition to the direct effects on macrophages, CAFs can also indirectly promote macrophage polarization. CAF‐derived Chi3L1 can upregulate the expression of Th2 cell‐related factors (IL‐10, IL‐4, Gata3, IL‐13), which indirectly promotes M2 polarization and shapes the immunosuppressive microenvironment in BC.^108^

TA‐MSCs have also been shown to promote TNBC metastasis through interactions with TAMs. Li et al.^62^ found that fibroblast activation protein alpha (FAPα) was overexpressed in TA‐MSCs, which promoted TA‐MSCs to secrete CCL2. Overexpressed CCL2 induced the recruitment of CCR2+ TAMs and M2 polarization, thus facilitating TNBC lung metastasis. A FAPα‐activated vinblastine prodrug can suppress CCR2+ TAM recruitment and polarization, and thus inhibit pulmonary metastasis of orthotopic TNBC.

In addition to cells in TME, tumor matrix can also influence the polarization of TAMs. According to a recent study, matrix stiffness affected the accumulation of M2‐type macrophages in BC. Elevated matrix stiffness increased CSF1 expression in BC cells and induced a significantly higher concentration of M2‐type macrophages in TME.^109^

In summary, the molecular mechanism of TME on macrophage polarization is complex and comprehensive. More studies are required to clarify the connection between TME and TAMs and to find more individualized therapeutic targets.

#### 3.3.2 Signaling pathways in TAMs

Macrophage membrane proteins are important features for identifying subtypes of TAMs and targets for reprogramming TAMs in BC. Elevated expression of plasma membrane‐bound sphingomyelin synthase 2 (SMS2), involved in maintaining the Sphingomyelin (SM) level on the macrophage membrane, is associated with an enriched TAM signature and a worse prognosis in TNBC patients.^110^ SMS2 inhibitor alleviates macrophage M2 polarization and enhances an abundant amount of CD8+ T cell infiltration. Integrin β3 is over‐expressed on the cytomembrane of TAMs with M2‐like characteristics.^111^ With the intervention of integrin β3 inhibitor, the M2 polarization of TAM is inhibited and the M1/M2 ratio of TAM is upregulated. In addition, the “macrophage receptor with collagenous structure” (MARCO), which is a pattern‐recognition receptor of the class A scavenger receptor family, was identified as a gene highly expressed in the TAMs and defined a subtype of TAMs with an M2‐like signature.^112^ Using anti‐MARCO mAbs to target these TAMs, anti‐tumor activity was induced in both the primary and metastatic breast carcinoma. Furthermore, macrophage annexin 1 (ANXA1), induced by tumor cell‐derived CCL5, is important in regulating polarization and activation of M2‐type macrophages.^113^ The absence of ANXA1 enhanced polarization shift to the M1 phenotype.

Cytokines or pathological signals from TME act on the surface or intracellular receptors of TAMs, resulting in stimulating a series of signaling pathways and promoting TAM polarization. These signaling pathways are extremely important for reprogramming TAMs. NF‐κB, which is involved in the transcription of CD4+ Th1 cytokines, has emerged as a central regulator of TAM function.^114^ Activation of NF‐κB in macrophages can lead to either an anti‐tumor phenotype or a pro‐tumor phenotype. In the early stage of lung metastasis of BC, activation of NF‐κB in macrophages leads to a shift to anti‐tumor phenotype in the lung and results in the reduction of lung metastasis.^115^ Some therapeutic drugs, such as baicalein, extracts of cordyceps sinensis, and cabazitaxel were demonstrated to inhibit BC growth by activating the NF‐kB signaling pathway and polarizing macrophages toward the M1 phenotype.^116^, ^117^, ^118^ Notably, the activation of NF‐κB induced by ROS accumulation promotes PD‐L1 transcription in macrophages, resulting in immunosuppressive phenotypes.^119^ M‐CSF secreted by tumor cells activated the expression of VEGF through stimulation of the NF‐κB pathway in TAMs, contributing to angiogenesis and BC progression.^120^ The regulation of macrophage function by NF‐κB is extremely complex and may be related to tumor stage and induction factors. Further studies are needed to target the NF‐κB pathway in TAMs.

Toll‐like receptors (TLRs) are a class of important molecules involved in nonspecific immunity and exert an enormous function on TAM polarization. There is increasing evidence showing that TLR4 could shift TAMs to the M1‐type and increase the expression of pro‐inflammatory cytokines.^121^ TLR4 mediated the repolarization of TAMs induced by therapeutics.^122^ Paclitaxel (PTX) suppressed tumor growth by impairing M2 polarization and reprogramming TAMs to an M1 phenotype through TLR4/NF‐κB pathway.^121^, ^122^ Similarly, anemoside A3 activates M1‐type polarization of TAMs via TLR4/NF‐κB/MAPK pathway to repress BC progression and angiogenesis.^121^ Stimulation of TLR7, which expresses in lysosomes of macrophages, also leads to the production of pro‐inflammatory cytokines, enhances the ratio of M1/M2 macrophages and increases the infiltration of CD8+ T cells. As a result, it prevents tumor growth and metastasis.^123^ Given the fact that TLR converts macrophages to an anti‐tumor phenotype, TLR agonists have been developed for cancer therapy.^124^

It is demonstrated that phosphorylation and dephosphorylation of STAT play a critical role in the regulation of TAM polarization. The phosphorylation of the key transcription factor STAT3, serving as a target of the IL‐6 receptor beta (glycoprotein 130, gp130) or TFEB in macrophages, mediated M2 polarization and promoted tumor cell proliferation and migration.^94^, ^125^ Similarly, enhanced STAT6 activity by phosphorylation, acetylation, or O‐GlcNAcylation modification, promotes macrophage polarization to an M2 phenotype.^126^, ^127^ The STAT6 pathway is critical to IL4‐induced M2‐type macrophages.^122^ The Hedgehog (Hh) pathway is a pivotal signaling pathway involved in driving TAMs to M2 phenotype and contributes to tumor growth in consequence.^128^ Further research has found that Hh‐induced M2 polarization is thought to be mediated by STAT6. Hh inhibitor, Vismodegib, accordingly governed the M2 state of macrophages.

As well, the PI3K/AKT/mTOR signaling pathway participates in the transformation from M1‐type to M2‐type macrophages and has been considered as a promising target. Selective inhibition of P13K/AKT/mTOR in TAMs can decrease pro‐tumor macrophages and increase M1‐type macrophages.^116^, ^129^, ^130^, ^131^ MAPK signaling pathway has great effects on the regulation of macrophage polarization. Blocking ERK or JNK signaling pathways is an effective method for reprogramming macrophages.^93^, ^113^ According to research findings, autophagy in TAMs was found to decrease the proportion of tumor‐promoting macrophages via the ROS/ERK and mTOR signaling pathways.^132^ Autophagy inducer can be used to inhibit the polarization of TAMs to M2‐type macrophages.

In summary, activating pro‐inflammatory signals or blocking anti‐inflammatory signals is an important measure to reprogram TAMs. Compared with the depletion of TAMs and inhibition of TAM recruitment, reprogramming macrophages has unique advantages. It not only reduces immunosuppressive TAMs but also increases pro‐inflammatory macrophages. Meanwhile, due to the avoidance of significant reduction of macrophages, the strategy has little effect on normal tissues. These advantages allow reprogramming macrophages to produce more pronounced therapeutic effects with fewer side effects. We need to further study the molecular mechanism of TAM polarization in order to provide more options for personalized targeted therapy in patients with BC.

### 3.4 Increasing phagocytosis

TAMs, as a kind of natural immune cells, have the ability to phagocytize tumor cells. However, tumor cells can evade the phagocytosis of TAMs through abnormally expressed signals. Enhancing macrophage phagocytosis is a vital factor to inhibit tumor growth. One of the mechanisms that induce macrophages to engulf cancer cells is to increase the "eat me" signal. Calreticulin (CALR) serves as a phagocytosis signal for macrophages.^133^ CALR is expressed on the tumor cell membrane and promotes phagocytosis of macrophages by interacting with low density lipoprotein receptor‐associated protein 1 on macrophages.^134^ Up‐regulation of CALR has emerged as a potential therapeutic mechanism by stimulating phagocytosis. Another mechanism is blocking the "don't eat me" signal. CD47 is overexpressed on BC cells, which inhibits macrophage phagocytosis through binding itself to its receptor, SIRPα.^135^ Targeting the CD47 / SIRPα axis not only blocks innate immune but also causes T‐cell activation.^136^, ^137^ Monotherapy by CD47 blockade leads to a reduction in tumor growth and an increase in OS.^138^ In addition, CD24 expressed on BC can also promote immune evasion through its interaction with Siglec‐10, which is expressed by TAMs.^29^ Ablation of either CD24 or Siglec‐10, as well as blockade of the CD24 / Siglec‐10 interaction, is a promising strategy for cancer immunotherapy. Moreover, programmed cell death protein 1 (PD‐1), an immune checkpoint receptor, was overexpressed on the surface of macrophages and negatively correlated with phagocytosis.^30^ Blocking PD‐1 / PD‐L1 would increase the phagocytosis of macrophages.

## 4 CHIMERIC ANTIGEN RECEPTOR (CAR)‐MACROPHAGE THERAPY

Recently, cell‐based immune therapy has developed rapidly in the treatment of malignant tumors. Given the success of CAR‐T therapy, researchers are increasingly paying attention to the antitumor potential of CAR‐macrophages (CAR‐M). Compared with CAR‐T cells, CAR‐Ms have a special advantage in the treatment of solid tumors due to the fact that macrophages are more likely to infiltrate into TME.^139^ CAR‐Ms are designed to contain an extracellular antigen‐recognition domain, a hinge domain, a transmembrane domain, and one or more cytoplasmic signaling domains.^140^ The design of the extracellular antigen‐recognition domain and cytoplasmic signaling domain is significant for the functions of CAR‐Ms. The extracellular antigen‐recognition domain is responsible for recognizing the target antigens overexpressed on other cells, such as CD19, CD22, and HER2.^141^, ^142^ The cytoplasmic signaling domains, for instance, FCγR and CD3ζ, are involved in signal transduction and immune cell activation.^141^, ^142^ Therefore, it is extremely important to design different extracellular antigen‐recognition domains and cytoplasmic signaling domains to achieve various anti‐tumor effects of CAR‐M.

A variety of extracellular antigen‐recognition domains and cytoplasmic signaling domains can be modified to target different cells and enhance the antitumor function of macrophages. Some progress has been made in animal models by using macrophages modified with specific CAR to improve phagocytosis, antigen presentation, and TME activation. Morrissey et al.^141^ designed CARs for phagocytosis (CAR‐Ps) to engineer murine macrophages. The engineered macrophages can target multiple extracellular ligands (CD19 and CD22) and combine multiple intracellular signaling domains (Megf10, FCγR, and CD3ζ). This CAR‐P strategy has been shown to promote specific phagocytosis. Note that, the portion of the CD19 cytoplasmic domain (amino acids 500 to 534) was fused into this structure to activate PI3K signaling. The co‐stimulatory intracellular domain significantly enhances phagocytosis, which can be extensively involved in designing CAR. In addition to enhanced phagocytosis, CAR‐modified macrophages can also promote macrophage polarization and T cell infiltration. Klichinsky et al.^142^ delivered an anti‐HER2 CAR with CD3ζ intracellular domain to human macrophages by a replication‐incompetent chimeric adenoviral vector (Ad5f35). The human anti‐HER2 CAR‐Ms have the ability of antigen‐specific phagocytosis of HER2+ tumor cells, resulting in reduced tumor load and prolonged survival period in the murine ovarian cancer model. Moreover, since Ad5f35 activated the macrophage inflammasome, the CAR‐Ms not only exhibited the M1 phenotype but also converted M2‐type to M1‐type macrophages. Zhang et al.^143^ also designed a CAR targeting HER2 for macrophages, which triggers the internal signaling of CD147 and activates MMPs to degrade the matrix. This CAR‐M showed the ability to boost anti‐tumor T cell infiltration and inhibit tumor cell growth in the murine TNBC model. Moreover, Niu et al.^144^ engineered a family of CAR‐Ms which target CCR7+ immunosuppressive cell population by CCL19 modification and trigger tumor cell cytotoxicity by the cytosolic domain from Mer receptor tyrosine kinase (MerTK). The above studies are directly modified macrophages. However, long‐term cultured macrophages in vitro are not suitable for clinical application because of altered gene expression. In order to solve this problem, Zhang et al.^145^ developed CAR‐expressing induced pluripotent stem cells (iPSCs) ‐induced human macrophages (CAR‐iMac), which contain an anti‐CD19 extracellular domain. CAR‐iMac exhibits antigen‐dependent phagocytosis and antitumor capability in vivo.

Engineered CAR‐Ms are a promising therapeutic approach for BC, especially for HER2+ cancers. However, CAR‐M therapy is still in its early stages. One Phase I trial of CAR‐M to target HER2‐overexpressed solid cancers is currently ongoing (NCT04660929). It is expected that the results of relevant clinical trials will provide valuable guidance for safe and effective CAR‐M therapies.

## 5 NDDSS AGAINST TAMS

In the last few years, a substantial number of drugs have been developed to attack TAMs, whereas the clinical applications remain limited due to the shortcomings of TAM‐specific agents, such as poor solubility, rapid metabolism, non‐selectivity, and off‐target effect. Fortunately, the rising NDDSs have opened up bright prospects for overcoming the above barriers. Targeting TAMs using NDDSs is an extremely attractive treatment due to the phagocytosis of TAMs. The following subsections will review recent advances of NDDSs against TAMs in BC, including the delivery of TAM‐specific immunotherapeutic agents to TAMs, the delivery of nucleic acid therapeutics to TAMs, and combination therapy.

### 5.1 Delivery of TAM‐specific immunotherapeutic agents

Similar to chemotherapeutic or immunotherapeutic drugs, many TAM‐specific agents have poor biocompatibility, low drug concentration in tumor tissues, and serious adverse reactions. One application of NDDSs is to avoid these problems and improve their efficacy. Table 2 summarizes NDDSs for delivering TAM‐specific immunotherapeutic agents.

**TABLE 2:** Delivery of TAM‐specific immunotherapeutic agents in BC

Ligands/ Receptors	Therapeutic agents	Delivery systems	Signaling pathways	Tumor cell lines	References
Fc/ Fc γ receptor	IgG3 Fc	MSN‐Fc	NF‐κB	4T1	^146^
HA/ CD44	Methotrexate	PeiPLGA‐MTX NPs	STAT3/ NF‐κB	4T1	^147^
Galactomannan/ CD206	HC	PSGM‐HCNP	STAT3	4T1	^148^
	RSL3	MIL88/RSL3	STAT1, IRF5, NF‐κB	4T1	^151^
	Imiquimod	PLGA‐ION‐R837@M	IRF5, TLR7	4T1	^149^
Mannose/ CD206	HA	Man‐HA‐MnO_2_	TLR4	4T1	^150^
mUNO/ CD206	Resiquimod	LNPs	TLR7/8	4T1	^124^
FA/ FR	TLR7a/PI3Ki	FA‐TLR7a/FA‐PI3Ki	TLR7 or PI3K	4T1	^123^
Mannose/ CD206	3‐MA	PHNPs@DPA‐S‐S‐BSA‐MA@3‐MA	P13K	MDA‐MB‐231	^152^
αvβ3‐mimetic antagonist/ integrin	MI3‐PD	αvβ3‐MI3‐PD NP	c‐MYC	4T1	^153^
GRP78P/ GRP78 proteins	IL‐12	TRN	IL‐12	4T1	^154^
Dextran/ CD206	BLZ‐945	DH@ECm	CSF1R	4T1	^156^
GBI‐10/Tenascin‐C	ZA	Apt@(DGL‐ZA)n NPs	NF‐κB	4T1	^158^
DNA scaffolds/ Scavenger receptor	E64	E64‐DNA	Cysteine proteases	E0771	^159^
	BLZ‐945+ selumetinib	DSN	CSF1R and MAPK	4T1	^165^
MP/ CD206 + TLR4 + TLR2	MP + CQ	MP‐ss‐PLGA@CQ	NF‐κB and TFEB	4T1	^164^
	CSF1R inhibitor +SHP2 inhibitor	DNTs	CD47 and CSF1R	4T1	^166^
	Sorafenib+aCD47	DLG	CD47 and MAPK	4T1	^167^
aCD47	CALR+ aCD47	SNPA_CALR&aCD47_	CD47 and CALR	4T1	^134^

It can be seen that single‐drug loaded NDDSs are mainly used to deliver agents that re‐polarize M2‐type macrophages to an anti‐tumorigenic M1 phenotype.^123^, ^124^, ^146^, ^147^, ^148^, ^149^, ^150^, ^151^, ^152^, ^153^, ^154^ For example, Hydrazinocurcumin (HC), a pyrazole derivative of curcumin (Cur), which has poor stability, bioavailability, and pharmacological activities, was reported to exert antitumor ability through re‐polarization of TAMs.^155^ Kumari et al.^148^ developed self‐assembled amphiphilic PEGylated galactomannan (GM) NPs loaded with HC (PSGM‐HCNPs) to target CD206. Note that, the findings indicated that M2‐like RAW264.7 cells treated with PSGM‐HCNPs exhibited elevating ROS levels, decreasing CD206 and Arg‐1 expressions and increasing pro‐inflammatory cytokine secretion, implicating that PSGM‐HCNPs re‐polarize TAMs from anti‐inflammatory to pro‐inflammatory phenotype. RSL3 is an iron death activator, which can enhance iron‐dependent lipid peroxidation in cancer cells and macrophages, destroy mitochondrial membrane structure, and lead to M1 phenotype polarization.^151^ However, the hydrophobicity of RSL3 limits its clinical application. Gu et al.^151^ designed an iron‐based metal‐organic framework nanoparticle (MIL88B) that can load RSL3. RSL3‐loaded MIL88B impaired mitochondrial functions, forcing the macrophage to undergo glycolytic metabolism and ultimately inhibiting tumor growth and metastasis.

In addition, agents which can deplete TAMs have been delivered to tumor tissues through nanoparticles in vivo for tumor treatment. Wang et al.^156^ developed an erythrocyte‐cancer cell hybrid membrane camouflaged dextran‐g‐poly (histidine) copolymer (DH@ECm) to deliver BLZ‐945, a hydrophobic drug with the ability of CSF1R inhibition, to M2‐type macrophages. Hybrid membrane not only has the ability of immunity camouflage but also has tumor targeting ability to tumor tissues. In TME, dextran was exposed to be bound to CD206 expressed on TAMs. DH@ECm possessed the best antitumor activity with an inhibition rate of 64.5%, which is three times that of the free drug. Nitrogen‐containing bisphosphonates (N‐BPs), such as zoledronic acid (ZA), are used to deplete TAMs and reverse the polarization of TAMs. However they have a high affinity with bone, leading to lower drug concentrations in tumors.^157^ Guo et al.^158^ encapsulated ZA with synthesized Dendrigraft poly‐L‐lysines (DGLs) to overcome the shortcoming. The ratio of M1 (CD16/32+) / M2 (CD206+) macrophage was significantly increased after the treatment of (DGL‐ZA)n NPs compared with ZA, which indicated the repolarization effect of the (DGL‐ZA)n NPs against M2‐type macrophages.

There is evidence suggesting that lysosomal cysteine protease activity in M2‐type macrophages is induced to degrade tumor antigens and hinder antigen cross‐presentation.^159^ The small molecule cysteine protease inhibitor E64 can be used to treat TAMs. However, E64 is difficult to penetrate into cells, which may limit its entry into lysosomes. The DNA scaffold can be used as a special nanodrug carrier. Cui et al.^159^ conjugated E64 to a 38‐base pair DNA duplex in order to localize E64 to the lysosomes of TAMs through scavenger receptors. E64‐DNA was intravenously delivered to target TAMs and attenuate lysosomal cysteine protease activity for the purpose of activating CD8+ T cells, which results in a good control of tumor burden without changing the TAM phenotype.

Some nanocarriers designed for drug delivery have the ability to regulate TAMs, resulting in synergistic interactions with TAM‐specific drugs. Xie et al.^160^ synthesized one cationic polysaccharide spermine modified pullulan, which not only facilitated the repolarization of TAMs by upregulating TLR but was also performed as a nanocarrier to deliver drugs or RNA. Ferumoxytol, an iron oxide nanoparticle approved by the Food and Drug Administration, can polarize M2‐type macrophages to M1‐type macrophages to activate the anti‐tumor response.^161^ Nevertheless, the response induced by ferumoxytol alone was limited and could not inhibit tumor growth significantly. Li et al.^152^ developed mannose‐bound porous hollow iron nanoparticles (PHNPs), loading a P13K γ small molecule inhibitor (3‐methyladenine, 3‐MA). With PHNPs@DPA‐S‐S‐BSA‐MA@3‐MA treatment, the repolarization of TAMs and higher therapeutic efficacy were achieved.

One of the advantages of NDDSs is that they can co‐deliver multiple drugs. Compared with single TAM‐special drugs, the combinational delivery of two TAM‐special drugs with different targets could produce a better therapeutic effect. Chloroquine (CQ) is an effective antimalarial drug and reprograms TAM metabolism from oxidative phosphorylation to glycolysis.^162^ Polysaccharides and CQ re‐educate TAMs via different signaling pathways. Thus, the co‐delivery of polysaccharides and CQ may play a synergy role.^163^ Yang et al.^164^ developed a hydrophobic poly(lactic‐co‐glycolic acid) (PLGA) segment loading Lepidium meyenii Walp. (maca) polysaccharide (MP) and CQ. MP‐ss‐PLGA@CQ was selectively absorbed by M2‐type macrophages rather than tumor cells in the 4T1–M2 co‐culture model, resulting in the highest proportions of M1‐type macrophages and higher inhibitory effect in situ and distant metastasis. Besides, therapeutic inhibition of CSF1R and its downstream MAPK signaling could effectively re‐polarize M2‐type macrophages to an anti‐tumorigenic M1 phenotype. Ramesh et al.^165^ designed dual‐kinase inhibitor‐loaded supramolecular nanoparticles (DSNs) to deliver CSF1R inhibitor and MAPK inhibitor together. Expectedly, the co‐delivery of CSF1R inhibitor and MAPK inhibitor resulted in re‐polarizing M2‐type macrophages to an anti‐tumor M1 phenotype and more robust tumor suppression than single delivery in an aggressive 4T1 tumor model.

Increasing the "eat me" signal or inhibiting the "don't eat me" signal to enhance the phagocytosis of TAM is one of the anti‐tumor strategies targeting TAMs. However, single immunotherapeutic agents cannot produce the best therapeutic effect, and the systemic immune system can be activated by immunotherapy, resulting in significant adverse reactions. Zhang et al.^134^ designed an NDDS in which CALR and aCD47 were covalently conjugated onto the surface of azide‐modified silica NPs (SNPAs). Flow cytometric analysis indicated that SNPA _CALR&aCD47_ increased the percentage of tumor cell‐ingested macrophages by more than twofold compared with CALR+aCD47+ SNPA or SNPA _CALR_ + SNPA_aCD47_. After being intratumorally injected in an orthotopic 4T1 tumor model, SNPA CALR&aCD47 exhibited a stronger antitumor efficacy across all the treatments. Furthermore, the combined delivery of CD47 antibodies and drugs that can deplete or reprogram TAMs also produced additive therapeutic effects.^166^, ^167^

### 5.2 Delivery of TAM‐specific nucleic acid therapeutics

In addition to the delivery of TAM‐specific immunotherapeutic agents, another potential method to regulate macrophage activity is TAM‐specific nucleic acid therapeutics by NDDSs. However, a number of challenges, including rapid degradation and off‐target effects, have hindered the clinical application of nucleic acid therapeutics.^168^ NDDSs have attracted more and more attention due to their advantages to enhance the stability and cellular uptake of macromolecules such as siRNA, shRNA, and miRNA. By now, a series of such delivery systems have been constructed for the delivery of TAM‐specific nucleic acid therapeutics in BCs. These systems reported in the literature are summarized in Table 3. In the following, the description and discussion of these results are provided.

**TABLE 3:** Delivery of TAM‐specific nucleic acid therapeutics in BC

Therapeutic agents	Delivery systems	Target genes	Tumor cell lines	References
siCCL18	NP‐180	*CCL18*	MDA‐MB‐231	^169^
siCCR2	CNP/siCCR2	*CCR2*	4T1	^170^
siMIF	Glucan‐based siRNA carrier	*MIF*	MDA‐MB‐231/ 4T1	^171^
siVEGF + siPIGF	PEG=MT/PC/siVEGF siPIGF NPs	*VEGF and PIGF*	4T1	^172^
VEGF inhibitor + siMED1	MT/PC/siV‐D NPs	*VEGF and MED1*	4T1	^173^
CRISPR‐RICTOR	CRISPR‐RICTOR‐Liposomes	*RICTOR*	4T1	^174^
miR‐125b	RLS/MNPs/miR‐125b	*IRF4*	4T1	^176^

SiRNA is a kind of double‐stranded RNA, which can cause the degradation of specific mRNA after transcription. The silencing of genes with siRNA has the potential to inhibit the development of tumors. Nevertheless, the rapid degradation and poor cellular uptake of siRNA are challenges for siRNA‐based therapy. Using NDDSs to deliver siRNA may be a promising method to overcome these defects of siRNA. Liang et al.^169^ developed 180nm nanoparticles which are composed of biodegradable poly (ethylene glycol)‐b‐poly (𝜀‐caprolactone) (PEG‐b‐PCL), poly (𝜀‐caprolactone)‐b‐poly (2‐aminoethyl ethylene phosphate) (PCL‐b‐PPEEA), and PCL homopolymer. These nanoparticles are able to load siCCL‐18 through charge absorption and deliver it to TAMs. As CCL‐18 is a significant factor secreted by TAMs and enhances the metastasis of BC, the carried siCCL‐18 could silence *CCL‐18* and thus inhibit BC's metastasis. The experimental results show higher cell uptake of the siCCL‐18‐loaded‐nanoparticles than the pure siCCL‐18, which verifies the effectiveness of the developed nanoparticles in inhibition against BC cell migration. Rafael et al.^170^ developed positively charged PEG–PLA nanoparticles for delivering CCR2 siRNA to inflammatory monocytes. CCR2, a major transmembrane protein of TAMs, was reported to be associated with macrophage recruitment. The CCR2‐siRNA delivery system mediated the blockade of macrophage recruitment and switched the immunosuppressive environment to an immunostimulatory environment. Zhang et al.^171^ developed a glucan‐based siRNA carrier system (BG34‐10‐Re‐I) and demonstrated that the BG34‐10‐Re‐I can effectively assemble siMIF into tumor cells and TAMs. The reduction of MIF in TAMs resulted in a significant reduction of factors that marks M2 polarization, meanwhile, the reduction of MIF in tumor cells resulted in a significant decrease of tumor cell proliferation and an increase of tumor cell apoptosis.

In addition, the co‐delivery of two siRNAs to TAMs may produce a synergistic anti‐tumor effect, and become a more efficient treatment. Song et al.^172^ co‐delivered VEGF siRNA (siVEGF) and PIGF siRNA (siPIGF), which were up‐regulated in both bulk tumor cells and TAMs, using polyethylene glycol (PEG) and mannose doubly modified trimethyl chitosan (PEG = MT) along with citraconic anhydride grafted poly (allylamine hydrochloride) (PC)‐based nanoparticles (NPs) (PEG = MT/PC NPs) with dual pH‐responsiveness. PEG = MT/PC/siVEGF/siPIGF NPs exhibited stronger inhibition of tumor growth and lung metastasis compared to a single delivery. Similarly, the co‐delivery of VEGF inhibitor and siMED1 also showed a better inhibitory effect on BC.^173^

Moreover, some studies pay more attention to permanently modulating TAMs at the molecular level. The CRISPR system is a possible method of permanently modulating macrophage polarization. Compared to siRNA, CRISPR has lower off‐target efficiency. Leonard et al.^174^ designed CRISPR‐RICTOR‐Liposomes, which can knock down RICTOR. Since RICTOR is an adapter protein in the mTORC2 complex, silencing RICTOR can block macrophage polarization to the M2 phenotype. The application of CRISPR‐RICTOR‐Liposomes reduced the proportion of M2‐type macrophages and increased the efficacy of PTX in BC.

Recently, several miRNAs have been explored as novel therapeutic targets, showing regulatory effects on TAMs and tumor cells. MiR‐125b has a TAM regulatory function, and its overexpression drives TAM adaptation to activated morphology and stimulates T cell activation.^175^ As with other gene transfer processes, efficient delivery of miRNAs remains a major challenge due to the rapid degradation of genes by ubiquitous RNases. Hu et al.^176^ synthesized a parallel and cascade control system, composed of cationic lipopeptides with an arginine‐rich periphery (RLS) and anionic magnetic nanoparticles (MNPs) for fleet transfection of miR‐125b. MNPs have high transfection efficiency, contributing to inhibiting tumor growth and metastasis by inducing polarization to M1‐type macrophages in breast tumors.

### 5.3 NDDSs for combination therapy

It is useful to regulate the function of TAMs for tumor inhibition, whereas a single TAM‐special treatment may not permanently control the growth of tumors. In addition, monotherapy to kill tumor cells has limited effect and often causes drug resistance of tumor cells. Therefore, combination therapy targeting tumor cells and TAMs has attracted much attention. Combining TAM treatments with other therapies, such as chemotherapy, immunotherapy, and phototherapy, is a promising therapeutic strategy for breast tumor therapy. Through the nanosized drug delivery system, multiple therapeutic agents with various anti‐tumor mechanisms can be delivered to tumor cells and TAMs at the same time and achieve better anti‐tumor effects.

#### 5.3.1 Co‐delivery of TAM‐Specific agents and chemotherapeutics/immune checkpoint inhibitors

Chemotherapy is one of the important methods for the treatment of BC. However, due to the limited accumulation of drugs in tumor tissues, it could produce toxicities and poor therapeutic qualities. TAMs play an important role in tumor response to chemotherapy, and the tumor‐promoting and immunosuppressive effects of TAMs limit the effect of chemotherapy.^34^ The combination of TAM‐specific agents and chemotherapy is extremely advantageous. Recently, many researchers have reported the successful development of co‐delivery systems loaded with traditional chemotherapeutic drugs and TAM‐specific agents for BC therapy (Table 4). Several nanoparticles, such as fucoidan, zymosan, and Fe_3_O_4_, have been reported to have the ability to deplete or reprogram TAMs.^177^, ^178^, ^179^, ^180^, ^181^ It is advantageous to use them as carriers to deliver chemotherapy drugs to BC tissues since they not only exert the killing effect of chemotherapy on tumor cells but also improve innate immunity.

**TABLE 4:** Co‐delivery of TAM‐specific agents and chemotherapeutics/immune checkpoint inhibitors in BC

Ligands/ Receptors	Therapeutic agents	Delivery systems	Therapeutic strategies targeting TAM	Tumor cell lines	References
Mannose/ CD206	DOX	DOX‐AS‐M‐PLGA‐NPs	Depletion	M‐Wnt	^177^
	DOX	PEI‐FCD‐DOX NPs	Reprogramming	4T1	^178^
	DOX	PEG‐PEI‐ZYM‐DOX NPs	Reprogramming	4T1	^179^
HA/ CD44	DOX	Fe_3_O_4_–DOX–HA	Reprogramming	4T1	^180^
AT_pep_ / europilin‐1 + Fc receptor	DTX	ATpep‐NPs‐DTX	Phagocytosis	4T1	^181^
iRGD + ApoE / integrin + LDLR	MMC + DOX	iRGD‐DMTPLN	Depletion	MDA‐MB‐231	^182^
	Indoximod + DOX	DOX/IND@NPs	Depletion	4T1	^164^
	HCQ + DOX	AuNPs‐D&H‐R&C	Reprogramming	MCF‐7/ADR	187
Chondroitin sulfate/ CD44	Imiquimod + DOX	PLGA	Reprogramming	4T1	^184^
FA/ Folate receptor	DHA + PTX	PTX/DHA‐FA‐LNs	Reprogramming	MCF7	^185^
	aCD47 + PTX	PTX‐Ilips	Phagocytosis	MDA‐MB‐231	^186^
	Pexidartinib + aPD‐1 antibody	PLX‐NP‐P‐aPD‐1@Gel	Depletion	4T1	^188^
	Pt(IV)+ CQ + ^D^PPA‐1	Pt(IV)/CQ/PFH NPs‐^D^PPA‐1	Reprogramming	4T1	^189^

Moreover, NDDSs are used to co‐deliver chemotherapeutics and TAM‐specific agents to achieve synergistic therapeutic effects.^177^, ^182^, ^183^, ^184^, ^185^, ^186^ For instance, Xie et al.^183^ developed furin‐instructed aggregated gold nanoparticles to co‐deliver doxorubicin (DOX, a conventional chemotherapeutic drug) and HCQ (an inhibitor of autophagy), producing AuNPs‐D&H‐R&C particles. HCQ activated the p53‐dependent apoptosis pathway and increases the tumor cell's sensitivity to DOX. Moreover, HCQ enhanced the NF‐κB nuclear translocation in TAMs and thus activates NF‐κB pathway, which re‐educates tumor‐promoting TAMs to anti‐tumor phenotype. The co‐delivery of HCQ and DOX improved antitumor effects. This observation inspires a regimen for the treatment of BC by combing chemotherapy and TAM reprogramming.

Even though numerous co‐delivery systems have been developed, the challenge associated with the delivery to cancer cells and TAMs respectively in a single NDDS has remained. Interestingly, Li et al.^184^ designed a localized drug delivery system, PLGA(H)‐DOX@M/R837, with a step‐by‐step cell internalization ability based on a hierarchical‐structured fiber device. The DOX‐loaded nanomicelles are encapsulated in the internal chambers of the fiber, which could first be internalized by tumor cells via binding to the overexpressed CD44 receptor to induce ICD. Next, the rod‐like microparticles can be gradually formed from long to short shapes through hydrolysis of the fiber matrix in the TME and selectively phagocytosed by TAMs when the length becomes less than 3μm. The TLR7 agonist imiquimod could be released from these short rod‐like microparticles in the cytoplasm to reprogram M2‐type TAMs. The tumor inhibition rate of the PLGA(H)‐DOX@M/R837 group reached 92.41%, which was higher than that of the single delivery group of DOX or TLR7. The sequential release of chemotherapeutic drugs and TAMs‐special agents in NDDSs achieved better therapeutic effects.

The immune microenvironment of malignant tumors is mostly in the state of immunosuppression. The abnormality of immune checkpoint protein is the main mechanism of immune escape in BC, especially in TNBC.^187^ Immune checkpoint inhibitors have recently become a focus of global attention and a “new hope” for cancer treatment. Immune cells, such as CD8+ T lymphocytes, attack tumor cells when PD‐1 or PD‐L1 is blocked by antibodies. However, the main obstacle to the clinical application of immune checkpoint inhibitors is the disorder of the immune system. Therefore, TAM‐specific agents and immune checkpoint inhibitors can be considered to co‐encapsulate in a single NDDS for improving both innate and adaptive immunity.^188^, ^189^ For example, platinum(IV) (Pt(IV), a chemotherapeutic agent), CQ, ^D^PPA‐1 (an anti‐PD‐L1 peptide), and perfluorohexane (PFH, ultrasonic contrast agent) were loaded in a pH/GSH dual‐sensitive nanoparticle.^189^ The Pt(IV)/CQ/PFH NPs ‐^D^PPA‐1 drug delivery system reversed immunosuppression in TME and displayed excellent anti‐BC efficacy.

#### 5.3.2 Co‐delivery of TAM‐specific agents and photosensitizers

In recent years, phototherapy, including photothermal therapy (PTT) and photodynamic therapy (PDT), has gradually become the main means of tumor treatment. For photothermal therapy, the light at a specific wavelength irradiates and heats up photothermal agents to kill tumor cells.^190^ In the case of photodynamic therapy, photosensitizers can produce large amounts of reactive oxygen species (ROS) which can kill tumor cells under specific light exposure.^191^ Compared with surgery, radiotherapy, and chemotherapy, phototherapy has the advantages of strong manipulation, precise target, and fewer side effects. Moreover, tumor cell fragments generated by phototherapy can act as tumor‐associated antigens and induce anti‐tumor immune responses. However this effect is not enough to alleviate the immunosuppression of TME and completely cure cancer.^192^ Therefore, multimodal treatment of phototherapy combined with TAM‐specific agents may have broad prospects in combating BCs.

It was reported that some photosensitizers, such as black phosphorus (BP), Mn, and Zn, can act as nanocarriers to deliver drugs.^193^, ^194^ Zhang et al.^194^ successfully developed a targeting BP nanoparticle loaded with PEGylated hyaluronic acid (HA). BP‐HA possessed better photothermal efficiency, ^1^O_2_ generation efficiency, and stability than BP. HA‐BP nanoparticles combined with 808 + 635 nm laser induced immune response and exhibited a valid anti‐tumor effect in vivo. Notably, BP‐HA could re‐educate TAMs from M2 to M1 phenotype, mainly due to the role of low molecular HA rather than laser irradiation.

A number of other nanomaterials cannot act as photosensitizers by themselves but can carry photosensitizers and TAM‐specific agents to produce synergistic therapeutic effects. Jian et al.^195^ investigated if the liposomal nanoparticles embedded in manganese dioxide (MnO2), hydrophobic photosensitizer (IR780), and ZA had a favorable effect on BCs. Lipo ZA/IR NPs generated O_2_ bubbles through MnO_2_ in response to H_2_O_2_ in TME, leading to the degradation of the liposomal membrane and causing the separation of ZA and IR780. Microcalcifications enable ZA to target TAMs, resulting in immunomodulation. LyP‐1 guides IR780 to target tumor cells for PDT with adequate O2 supply. It is worth noting that the large amount of ROS produced by PDT not only kills primary tumor cells but also induces immunogenic cell death (ICD) and induced polarization of pro‐inflammatory M1‐type macrophages.^192^ As expected, the synergistic effect of ZA and PDT could significantly improve the anti‐tumor ability. These findings provide appropriate implications and guidance for the design of photo‐immunotherapy.

In addition, the combination of immune checkpoint blockade and photothermal therapy provides a potential therapeutic approach. Zhao et al.^4^ built a stimuli‐responsive multifunctional nanoplatform (ZIF‐PQ‐PDA‐AUN), which encapsulated AUNP‐12 (a PD‐1 analog), PQ912(a CD47 inhibitor) and PDA (a photothermal conversion substance). The NDDS avoided the systemic side effects of immunomodulators and improved anti‐tumor efficacy by reshaping innate and adaptive immunity. Thermal ablation combined with the immune checkpoint blockade demonstrated its effectiveness in tumor suppression. Zhang et al.^196^ constructed a NIR‐triggered core‐satellite upconverting nanoparticle with Cur embedded (Cur–CSNPs). The ∼450 nm luminescence converted from the 980 nm light by UCNPs activated Cur to produce ROS and induced ICD. Moreover, Cur could repolarize TAMs from pro‐tumor M2 to anti‐tumor M1 phenotype via inhibiting STAT3 activity. The combination of Cur and PDT achieved the most effective antitumor effect among all groups.

### 5.4 Active‐targeting NDDSs

In the past decades, the enhanced permeability and retention (EPR) effect has become an important factor in the design of NDDSs and plays a key role in passive targeted delivery.^197^ However, the validity of the EPR effect in cancer patients has become the focus of debate. The EPR effect may be limited in some tumors with poor blood supply because of its association with tumor blood vessels. In addition, a part of NDDSs entering tumor tissues was swallowed by tumor cells or other non‐malignant cells in TME, reducing their enrichment in TAMs. Therefore, in order to improve the concentration of NDDSs in TAMs, it is necessary to design NDDSs with active targeting functions.

By now, receptors that are highly expressed on the cell membrane of M2‐type TAMs have been used to functionalize NDDSs, which can facilitate the targeted delivery of therapeutic drugs to TAMs through the interaction between ligands on the surface of NDDSs and receptors on the membrane of TAMs. For example, CD206 are M2‐type macrophage markers with high specificity, making it one of the most commonly targeted receptors for TAMs.^48^ Mannose receptors are type I transmembrane glycoproteins that recognize and bind specific carbohydrate molecules such as mannose, galactomannan, and dextran through an extracellular cysteine‐rich domain.^148^, ^152^, ^156^ For example, Li et al.^152^ developed a mannose‐linked porous hollow iron oxide nanoparticles to deliver the small molecule inhibitor of PI3K γ to TAMs. Notably, findings in vitro indicated that the mannose‐linked targeted nanoparticles (MA‐NPs) exhibited higher cellular uptake of 3‐MA in RAW 264.7 rather than MDA‐MB‐231 cells compared with mannose‐unlinked control nanoparticles, owing to the specific targeting capability to mannose receptors overexpressed on the membrane of M2‐type macrophages. Furthermore, MA‐NPs have a better effect on reprogramming M2‐type macrophages and suppressing tumor growth than mannose‐unlinked nanoparticles. These results demonstrate that MA‐NPs are a promising nanocarrier design for TAM‐targeted cancer treatment. In addition to carbohydrate molecules, the "mUNO" peptide is designed to specifically bind to mannose receptors. Figueiredo et al.^124^ demonstrated that carrying mUNO binding to mannose receptor significantly enhanced cellular uptake of lignin nanoparticles loaded resiquimod in M2‐type macrophages, and significantly suppressed tumor growth, providing a method potentially applicable to BC by targeting and reprograming M2‐type macrophages.

In addition to TAM‐specific surface biomarkers, some membrane proteins overexpressed on TAMs, such as CD44, FR, and scavenger receptors, have been used to enhance the targeting ability of NDDSs.^159^, ^180^, ^185^ Particularly, receptors overexpressed on both tumor cells and TAMs mediated the accumulation of NDDSs in two types of cells. It has long been recognized that CD44, a receptor of chondroitin sulfate and HA, was highly expressed in BC cells and TAMs.^180^, ^184^ CD44 is thought to be a common receptor for NDDSs targeting tumor cells and TAMs at the same time. For example, Gong et al.^180^ constructed Fe_3_O_4_ nanoparticles with HA modification (Fe_3_O_4_‐DOX‐HA) for delivery of DOX to CD44+ 4T1 tumor cells and TAMs. The results showed that the accumulation of Fe_3_O_4_–DOX–HA in the tumor was higher than that of Fe_3_O_4_–DOX, which was due to the targeting effect of HA. In addition, enhanced antitumor efficacy was achieved by using Fe_3_O_4_–DOX–HA.

Besides CD44, FR is also used as a target of NDDSs. FRs are cysteine‐rich cell‐surface glycoproteins that bind folic acid (FA) with high affinity to mediate cellular uptake of nanoparticles.^198^ Although expressed at very low levels in most tissues, FRs are expressed at high levels in tumor cells and macrophages and are therefore a potential target of NDDSs. Li et al.^185^ successfully developed FA‐modified lipid nanoemulsions (PTX/DHA‐FA‐LNs) for the co‐delivery of PTX and docosahexaenoic acid (DHA). The results of confocal laser scanning microscopy showed that the accumulation of PTX/DHA‐FA‐LNs in MCF‐7 cells and M2‐type macrophages was stronger than that of PTX/DHA‐LNs. Furthermore, PTX/DHA‐FA‐LNs exhibited higher cytotoxicity to MCF‐7 cells and the ability to regulate macrophage polarization. Folate modification provided the prospect of targeting tumor cells and TAMs for the treatment of BC.

The strategy of targeting different receptors on tumor cells and TAMs, respectively, is also one of the effective means for dual targeting of NDDSs. Two different ligands were modified on the surface of NDDSs, which could bind to the corresponding receptors overexpressed on the membrane of tumor cells and TAMs to increase the accumulation of NDDSs in these two types of cells. This approach can deliver different drugs to tumor cells and TAMs respectively, facilitating precise targeting of NDDSs. For example, Zhang et al.^182^ developed a terpolymer‐lipid hybrid nanoparticle (TPLN) system with co‐loaded DOX and mitomycin C (MMC). TPLN was conjugated cyclic internalizing peptide (iRGD), a polypeptide containing Arg‐Gly‐Asp, which was able to selectively recognize and bind to integrins highly expressed on the membrane of BC cells, and thus had the effect of targeting tumor cells. Meanwhile, TPLN had the ability to recruit apolipoprotein E (ApoE) and targeted TAMs via low density lipoprotein receptor‐mediated endocytosis. Integrins and LDLR‐mediated targeted delivery increased cellular uptake of TPLN in BC cells and TAMs.

Taken together, satisfactory results have been achieved in active‐targeting NDDSs for TAM‐targeted therapy. More and more targets of TAMs provide new methods for the active‐targeted delivery of NDDSs. However, it should be noted that, in addition to tumor tissues, there are also a large number of macrophages in other tissues, which can bind and phagocytose ligand‐modified NDDSs and reduce the accumulation of NDDSs in tumor tissues. Precise delivery of NDDSs may be further facilitated by a strategy that NDDSs first target tumor tissues and are then taken up by TAMs. Peng et al.^181^ designed a dual‐targeting nanoparticle (AT_pep_‐NPs) system loaded docetaxel (DTX) for the treatment of BC. AT_pep_, composed of a phagocytosis‐stimulating peptide‐tuftsin (T_pep_) and a substrate peptide‐alanine‐alanine‐asparagine (AAN), can be cleaved by legumain in TME and activated into Tpep, promoting endocytosis of tumor cells and TAMs through neuropilin‐1 or Fc receptor. The TME‐responsive nanoplatform effectively avoids nonspecific uptake of active‐targeting NDDSs during blood circulation.

### 5.5 Limitations and challenges of NDDSs

At present, more than ten kinds of nanomedicines have been approved for clinical application, and a large number of new nanomedicines are undergoing clinical trials. Although a variety of NDDSs have been found to improve antitumor efficacy in preclinical studies, some of those are still failed in clinical translation. In phase I trials, the majority of NDDSs have shown positive results, with a high success rate of approximately 94%.^199^ The success rate of phase II and III trials was significantly lower than that of phase I.^199^ Reasons for the failure in clinical trials include poor efficacy or adverse effects. A Phase II study, radiation therapy, paclitaxel poliglumex, and carboplatin in Stage III non‐small cell lung cancer, was closed early due to respiratory failure (NCT00352690). Immune‐related adverse effects are also one of the reasons for the failure. A phase I clinical trial of Liposomal Mir‐34 (MRX34) was terminated because 20% of the patients had serious immune‐related adverse reactions, and the result was not reported (NCT02862145). These adverse effects suggest that nanotechnology should be regarded as a double‐edged sword, and the biological effects of NDDSs should be fully studied before they are applied to the clinic.

There are still limitations in the clinical transformation of NDDSs against cancers, and better design solutions need to be explored. Firstly, a main limitation is the biocompatibility of NDDSs. Interactions between NDDSs and components in the blood can affect the function of NDDSs, with unpredictable consequences. Blood contains a large number of proteins that bind tightly to the surface of nanoparticles to form a "protein crown", which changes their physicochemical characteristics and stability. It has been reported that the formation of protein crowns attenuates the active targeting of NDDSs and regulates their enrichment in cells. ApoE, a protein‐crown component, mainly mediates MoS2 enrichment in liver Kupffer cells and spleen red pulp macrophages.^200^ In addition, the protein crowns can also regulate the mechanism of nanoparticle entry into cells. The protein corona shifts the way liposomes cross the cell membrane from energy‐independent membrane fusion to energy‐dependent endocytosis.^201^ A growing number of studies are exploring strategies to regulate protein crowns. It is reported that the hydrophilic of NDDSs can effectively regulate the composition of protein crowns. With the increase of the hydrophilic degree of NDDSs, the IgE adsorption area could be decreased.^202^ However, reducing protein adsorption could not eliminate protein crowns. The use of protein crowns to deliver drugs is a promising way to block protein adsorption. Coating nanoparticles with de‐opsonins, such as albumin, transferrin, and apolipoprotein, can reduce macrophage phagocytosis and prolong its circulation in the blood.^203^ After entering the body, most nanoparticles are taken up and cleared by macrophages in the liver or spleen, which hinders further delivery to the tumor tissue and weakens the therapeutic effect. Pegylation is one of the methods for NDDSs to avoid being removed by macrophages. However, this modification resulted in reduced uptake of nanoparticles by tumor cells and the production of anti‐PEG antibodies after multiple injections. Biomimetic NDDSs have attracted the attention of researchers.^204^ Delivery of drugs through cell membranes or exosomes is being studied to improve the biocompatibility of NDDSs and reduce the clearance by macrophages.^205^

Secondly, the active targeting capability of NDDSs needs to be improved. One of the advantages of NDDSs over free drugs is that they can target tumor tissue. Several active‐targeted NDDSs, such as BIND‐014, CALAA‐01, and SGT‐94, have been tested in clinical trials, and their accumulation in tumor tissues is significant.^199^ However, no NDDSs with active‐targeting TAMs have ever entered clinical trials. One of the main characteristics of TAMs is their heterogeneity, with M1 and M2 phenotypes. How to avoid the off‐target effects of TAMs‐targeting approaches is a key problem to be solved. The molecular type of BC is crucial for TAMs‐targeting therapy. TNBC has a high density of CD163+ TAMs compared to luminal A.^55^ Therapies targeting CD163+ TAMs may achieve better efficacy in TNBC than in luminal A. Furthermore, the application of single‐cell sequencing and multiple fluorescence in situ detection techniques to identify surface markers of TAMs is of great significance for precision nano‐based therapies. The accumulation of NDDSs in M2‐type macrophages can be increased by linking antibodies targeting M2‐specific surface markers. In particular, the simultaneous binding of multiple antibodies targeting TAMs to NDDSs may reduce off‐target effects. Moreover, the design of the TME response can reduce the targeting of NDDSs to macrophages in other tissues. As we have mentioned, ATpep‐NPs, which are cleaved by legumain in TME, can be selectively targeted to TAMs.^181^ Besides, the pH of TME is lower than that of normal tissues and blood, and this phenomenon can be used to design NDDSs to trigger TAM targeting in BC tissues.^189^ In a nutshell, NDDSs designed based on the heterogeneity of TAMs are expected to realize personalized treatments of patients with BC and improve their efficacy.

Finally, it is necessary to design and develop more combined drug delivery systems. Although NDDSs targeting TAMs have achieved certain therapeutic effects, the efficacy of targeting TAMs alone is limited. Through NDDSs, multiple therapeutic agents with various anti‐tumor mechanisms can be delivered to tumor cells and TAMs at the same time. The advantage of such integrated NDDSs is the ability to control the location and sequence of drug release to achieve synergistic anti‐tumor effects. Combined delivery of multiple drugs can also reduce the dosage of each drug and reduce drug toxicity. In addition, the treatment of TAMs can also be combined with other treatments, such as PTT, PDT, and immunotherapy, to further improve the therapeutic effect.

## 6 DISCUSSION

TAMs, a prominent tumor‐associated noncancer cell type, play a critical role in breast tumor progression which includes proliferation, invasion, metastasis, immunosuppression, and angiogenesis.^44^ With an increased understanding of the biological characteristics of TAMs, an increasing number of strategies, including depleting macrophages, blocking recruitment, reprogramming to attain an anti‐tumor phenotype, and increasing phagocytosis, have been proposed to regulate TAMs and thus treat BCs. In this review, we systematically discuss the current development of anti‐TAMs strategies and NDDSs targeting TAMs. Such delivery systems can effectively overcome the deficiency of physicochemical properties of drugs, increase drug concentration in tumor tissues, reduce toxicity, and achieve multi‐drug combined delivery. There are still limitations in the clinical transformation of nanomedicine against TAMs. TAMs are heterogeneous, which include M1 and M2 phenotypes. The off‐target effects of TAMs‐targeting approaches are a key problem to be solved. To improve the ability to accurately target TAMs, three aspects should be considered in the design and application of NDDSs, which are the molecular type of BC, the application of detection technology, and the design of TME response. The pharmacokinetic characteristics, release sequence, and interaction of different drugs should be also all taken into account to achieve better therapeutic outcomes and facilitate the transition of TAM‐targeted NDDSs from experiments to clinical practice. In summary, NDDSs provide a promising therapeutic strategy for BCs through targeting TAMs, and further progress is expected to be made along with a deeper and deeper understanding of TAMs and NDDSs.

## AUTHOR CONTRIBUTIONS

**Cuiping Zhan:** Conceptualization (lead); data curation (lead); formal analysis (lead); investigation (lead); methodology (lead); resources (lead); validation (lead); visualization (lead); writing – original draft (lead); writing – review and editing (lead). **Xinzhi Xu:** Investigation (supporting); methodology (supporting); writing – original draft (supporting); writing – review and editing (supporting). **Jiangbo Shao:** Data curation (supporting); formal analysis (supporting); writing – original draft (supporting); writing – review and editing (supporting). **Ying Jin:** Funding acquisition (supporting); supervision (supporting); writing – original draft (supporting); writing – review and editing (supporting). **Chunxiang Jin:** Conceptualization (supporting); funding acquisition (lead); supervision (lead); validation (supporting); visualization (supporting); writing – original draft (supporting); writing – review and editing (supporting).

## FUNDING INFORMATION

This work was supported by Natural Science Foundation of Jilin Province (Grant Nos. YDZJ202201ZYTS643), National Natural Science Foundation of China (Grant Nos. 82003173), and Department of Finance of Jilin Province (Grant Nos. 2020SLZ46).

## CONFLICT OF INTEREST

The authors declare that the research was conducted in the absence of any commercial or financial relationships that could be construed as a potential conflict of interest.

## ETHICS STATEMENT

N/A.

## Data Availability

Data sharing is not applicable to this article as no new data were created or analyzed in this study.
